# Large disparities in HIV treatment cascades between eight European and high-income countries – analysis of break points

**DOI:** 10.7448/IAS.17.4.19507

**Published:** 2014-11-02

**Authors:** Alice Raymond, Andrew Hill, Anton Pozniak

**Affiliations:** 1 Department of Public Health, Imperial College London, London, UK; 2 Molecular and Clinical Pharmacology, Liverpool University, Liverpool, UK; 3 St Stephens Centre, Chelsea and Westminster Hospital, London, UK

## Abstract

**Introduction:**

Patients on antiretroviral treatment with undetectable HIV RNA levels have a significantly lower risk of clinical disease progression and onward HIV transmission. This study aimed to estimate and compare the percentage of all HIV-positive people who are diagnosed, are linked to care, are taking antiretroviral treatment and have undetectable HIV RNA, in eight European and high-income countries: the United States, the United Kingdom, France, the Netherlands, Denmark, Australia, British Columbia (Canada) and Georgia.

**Materials and Methods:**

For each country, the number of people in five key stages of the HIV treatment cascade was collected: 1. HIV infected, 2. Known to be HIV positive, 3. Linked to care, 4. Taking antiretroviral treatment, and 5. Having undetectable HIV RNA. Estimates were extracted from national reports [1–3], the UNAIDS database, conference proceedings [4] and peer-reviewed articles [5–7]. The quality of the estimates and reporting methods were assessed individually for each country, with selection criteria such as availability of nationwide database and routinely collected data. Treatment cascades were constructed using estimates from 2010 to 2012.

**Results:**

As shown in Table 1, the percentage of all infected people with undetectable HIV RNA ranged from 20% in Georgia to 59% in Denmark. Of the high-income countries, the United States has the lowest percentage of individuals with undetectable viral load (25% to median 52%), associated with the highest HIV incidence rate (15.30 per 100,000 to median 6.07 per 100,000). The pattern of the cascades differed between countries: in the United States, there is a fall from 66% to 33% (−33%) between linkage to care and start of antiretroviral treatment. However, in Georgia, the greatest loss in continuum was zat diagnosis, with 48% of undiagnosed HIV-positive individuals.

**Conclusions:**

There are great disparities among European and high-income countries in the percentage of HIV-positive individual with undetectable HIV RNA. Furthermore, the treatment cascades show different key break points, underlying inequalities in HIV care between countries.

**Table 1:** HIV treatment cascades in eight European and high-income countries

Countries	France	Netherlands	United States	United Kingdom	Australia	British Columbia	Denmark	Georgia
Number living with HIV	149,900	25,000	1,148,200	98,400	33,000	11,700	6500	4900
Percentage diagnosed	81	–	82	–	75	71	85	52
Percentage linked to care	>74	73	66	79	–	67	81	44
Percentage on ART	>60	59	33	67	35	51	62	26
Percentage with undetectable HIV RNA	52	53	25	58	32	35	59	20
